# Supplementary material for: Patterns of Intron Gain and Loss in Fungi
Source: PLoS Biol. 2004 Nov 30;2(12):e422. doi: 10.1371/journal.pbio.0020422 (PMC532390; doi:10.1371/journal.pbio.0020422)
Supplement: Table S1 — Also available at http://genes.mit.edu/NielsenEtAl/. (4.3 MB ZIP). [file pbio.0020422.st001.zip › NielsenEtAl/html/975.html]

AN5840.1.NCU05803.1.MG04710.1.FG09874.1


```
 CLUSTAL W (1.82) Multiple Sequence Alignments - Introns Inserted


Sequence 1: NCU05803.1	2692 aa
Sequence 2: MG04710.1	2678 aa
Sequence 3: FG09874.1	2864 aa
Sequence 4: AN5840.1	2788 aa
Alignment Length: 2923 aa
Number Identitical Residues: 1156 aa
Alignment Score (without introns) 59479


MG04710.1 	MTADVVNG--------DGPGQVDLVAIKQVLLSSSTKTRISQLRIVEDKISQNA1LDQAS
NCU05803.1	MAEDAAASPAPASTPSNAPADFDLVAAKAALTSSSTSARISQLRSIEEKLSQKA1LDRPT
FG09874.1 	----------------------------MCASPCSSPRPISPL-----TLHSAA~VDNTS
AN5840.1  	------------------------------------------------------~-----
          	                                                            

MG04710.1 	LSKLLGVLFLTHSSYADRPSRRAVQRCLVLLAKA-KEEKLLTALVA~AIRKESQKPTIAP
NCU05803.1	TLAVLKVLFWTHDFYHDRPSRTAVQKCLVALAQN-REPEILTPLVA~AIGQEIKKGAIAP
FG09874.1 	TARLLQLFFGTYAFYTDRQSRLSVQKCLVALISAGVDSKTIAPLIA~AVRKESQKQGIAP
AN5840.1  	-----MPPLSTQSPHSNRGSE----------------------ILG0TLKSDTETP----
          	        : *   : :* *.                      ::. :: .: :.     

MG04710.1 	ANAFVLLEWSSLLIEHLGETELWEKLGKELVLATSDCLEKCLQPQCRNNLGSSAQVVTRR
NCU05803.1	GSAFTLVEWCSLLVQNLAGTPLWEKFGKDLTLSLAEVLEKCCQPASRGTVSKSALVITRR
FG09874.1 	TNAFVLVEWCSLFMQHFDAS-LWDQFASDIILTDADALDKCHQSSARKSVAHSAIIVTRR
AN5840.1  	-----------------------LSVVLDIIALDARALETCLVAHPKPAVEKSAYRVTRR
          	                        ..  ::    :  *:.*  .  :  :  **  :***

MG04710.1 	AFRKLVSLEQTREKSITDAVQLLATKGAAPTAKYAPTLGVIAGVCARKDYGKPILEKLKP
NCU05803.1	GLRKLAAGDD-REKVIEEAVKALAAKGTQPTAKYAVLLGVIAGVCSRKPEAVPIIEKLKS
FG09874.1 	GLRKLFSSSDSSENRLSSSVDILASKSAQSTPRNAVLLGVIAGVSARKDHLRPVLDTLKP
AN5840.1  	ALRAVF-SDAWGDDAIKQSVQRLASN-ATTGQKNAPFLGVISGVSARLPNKKEVLAGEKK
          	.:* :   .   :. : .:*. **:: : .  : *  ****:**.:*      ::   * 

MG04710.1 	QFFGFYTREIIGSKTPVPSHFAEGLYDFFHSFVTLEDLGKEVTPALEKGLLRAPEIVLND
NCU05803.1	QYFTFYTREIVGSRTQVPPHQAAALSDFFSSFVTLEDLDKEVFPSLEKGLLRAPEVVLND
FG09874.1 	KYYEFFTREIIGSRIAVPEHVVLGLGDFFTSFAIPEELSKELIPALEKGLLRAPEVILGG
AN5840.1  	TLVAYYIKELVGTKAAPPAHIANGMSDFFESFITEADLTSDILPPLEKAILRSPEVVFCG
          	    :: :*::*::   * * . .: *** **    :* .:: *.***.:**:**::: .

MG04710.1 	LITPLVDALPSDYDLSSVLSKNLLKPLLANAKSTNASIRSGALTAFRKVVTRSADAETLG
NCU05803.1	LITPLVGALPQDFDLSAALHGKFVKPLLNNVKSSNAVIRSGAVTVFKGLAARSKDVALLE
FG09874.1 	VVTPLVRSLPESFDLSKVLEQSLLKPLLSNAKSTNPAIRAGALDAFRALVRRSNDTTSLE
AN5840.1  	LLPSLFSYLPTEIDLSEILYSRLLKHLLSSMKSNNPLIRQGALQSFESLLVKSKTESLLL
          	::..*.  ** . ***  *   ::* ** . **.*. ** **:  *. :  :*     * 

MG04710.1 	KATDEIVTPLKGGKIASAEQRAIHAEMLTALSTNEANATKICGAVPAVAGKEGNEPALVA
NCU05803.1	KVADEVLNPLKTGKLASADHRVLHSEMLVALVVSTGIANKIANGLPLVTGKEGNEAALSA
FG09874.1 	KVINEIATTLKGGKLASPDHRILHAQMLEATPLSTSSAEQVASAVATVAAKEGNEGALAA
AN5840.1  	KIAGEVIGPLKGNKITSPEQRAIYAQALSAVSPTTDISKDIIQGFVPVFTRESNEAALEQ
          	*  .*:  .** .*::*.::* :::: * *   .   : .:  ..  *  :*.** **  

MG04710.1 	ETKALLCSVKTLLNSGVEVPAAALDGYKKGLGDKKPGSRKIWVLSAGDLLLHSCSLPDSK
NCU05803.1	ETVALNASAVYLLNAGEEIPKPLADAYVKGLGEKKLPVRRTWILRAGDILYAFNKESQET
FG09874.1 	ETSALANAITFLLVQDGEVPKPVLDSVTKGLAEKKIPSRKYWLLRVGVILQSLAEAQS--
AN5840.1  	EIKAFAKHLTRLIQSKAKIPDDVVNTIVKGSAEKKIPFRRLWQLYIGQAIWQA--EPETL
          	*  *:      *:    ::*    :   ** .:**   *: * *  *  :       .  

MG04710.1 	IAP---KVQEVILPVLFDTYNEAVANPLAASQSGLIVGAYVTCTVAPSIFQTNKAAPIEA
NCU05803.1	LPANFIKFAEAVVTPLGTTFAEVVANPATAAQNGTVTGALVLCAVES-LIRRAESSTFEA
FG09874.1 	VSPGMVTFVEAVVPKMVTTLTEVTANAASAAQSGLVVGAYILTAVSPHIYRLLPGSTAES
AN5840.1  	ASNEIEPFVTKFVAKLEGIFKEVSSNPLPSAQNGALFTAYVYLALCG---RLSALEKFDK
          	 .     .   .:. :     *. :*. .::*.* :  * :  ::     :       : 

MG04710.1 	LLKKFSVSKQCLVWEPKPSFLLNPKLFSKLSTDEDFEWFFRALASVVAELP-EDGDSDVS
NCU05803.1	VYKKANIQKNSLAVNPKPSYLFNPRIYGKFA-DEDLKWLTSALTAVAPFLN-GVG-LPER
FG09874.1 	CLTKASVTKQSLSLDPKSSFLLSHRIYSKLTANEDLAWFARALSSVFQSLD-QNVDKHVA
AN5840.1  	AAWEENVG-HSMALQPKPSFLLNPKAYSKLASQAEVQWAVRTLAAVPSAAKFDDADAQTK
          	   : .:  :.:  :**.*:*:. : :.*:::: :. *   :*::*         .    

MG04710.1 	IAWAQALIYLISSPATGAQVRVDLCKRLSELYLSNPER-------ISTAVVGGMWSWLES
NCU05803.1	VAWAQAFIYVISSAATGPDVRRDAIDALSTLYAQSSAKAEGPSALLAETIVEGLWHWIEA
FG09874.1 	LAWSEAFIHLVTAHSVPAAIQQESSKLLSDLYVRNPRL-------VSSFIINGLWDHLEH
AN5840.1  	TAWAEAFIYAITAPGLHNSFRDESARNLSIVYKTNIGA-------IGRVITDALWSWILA
          	 **::*:*: ::: .    .: :    ** :*  .          :.  :  .:*  :  

MG04710.1 	VEEGEKDSAAALAKAENKH-LHLILRAICPSAQELPSKFTEEAGKEVLEAQLCSLLVLAR
NCU05803.1	TEAADKESAAALAKTGTSN-LHLVLKAICLKPEEYQKRAGAEPDKTKLESQMCSLLVLAK
FG09874.1 	SGSGDKE-----HSATAHN-LIQVVKSICLTPNEIS-KSGKSVAKEDLESQANSLLVLAR
AN5840.1  	FRTGEKESAPVSAGPDHLKFLHLVVKAICPAP---SSSESSEQTPAGLKSQLISLLLLAR
          	   .:*:::.    .   : *  ::::**  .    .    .     *::*  ***:**:

MG04710.1 	SDIVPRSSWIDLCLRSGLDPGQLATKHEKTLLQEVALR--SSFDQK0SDAIRTASYKAAV
NCU05803.1	SQLIPRASWIDLCLKVETDPGELARKYEQRLLDEIVNR--TLYEQK~SEAVRLAAYNAAA
FG09874.1 	SELIPRANWIDLCLRMELDPGNLVTKYQDELMAEIEAR--TSFDQK0VRVVKTAAYNAAA
AN5840.1  	PEMIPNAHWIDLCLRTGTDPGNLVREFSSDCMNQLVRVQEDPIQSK~VPQVDAAIWAAAG
          	.:::*.: ******:   ***:*. :...  : ::   ..   :.*    :  * : ** 

MG04710.1 	ELVFVAPEVMTPRIIQLIQHDLDTSELKDIGPLEAAIFRTPEGTTFVDVLAKKGTTVSTK
NCU05803.1	ELVFVAPETMTPRIVDLIQQNLKVSEVQSVGPLEAAIFRTPEGTAFVDVLAKKQNVVPNK
FG09874.1 	DLAFVAPETIIPLLLETLRRDLNAEQLQDIGPVEAAIYRTAEGTAFVDVLAKKSQQVLDK
AN5840.1  	DLAFVAPDVMVSQLVEQIKEDLDVSRVAHFTPTDAAIARTPEGTMFVDVLNTKSRQTFDK
          	:*.****:.: . ::: ::.:*....:  . * :*** **.*** ***** .*   .  *

MG04710.1 	GK-DADTLKWEAELRAQLAEKKGQ-PKKLSADEKAKVDAQLKKESAIRERVRGVAAKVMR
NCU05803.1	NDPNYHTLKWEEELREQIAQKKGV-QKKLTAEENAKVNAQLKKEAEIRESVRHVAANLLR
FG09874.1 	NKKDYDILKWEEELRSQLDKKKGQ-QKKLTPEENAKVNAQLKKESQIRQSITATVARLLR
AN5840.1  	NTKDYDILKWEEEMRAHLAQKQGEKPKKLTADEQAKVKAQLAKEAKIREDVLREVKRIER
          	.  : . **** *:* :: :*:* . ***:.:*:***.*** **: **: :   . .: *

MG04710.1 	GVGVIKSLATGPPTDATLWMGPAVNALLDAVDAGACLITGDAAPTAYLACADRVSSRLGS
NCU05803.1	GFGIVKALATGPPTDASRWMGPAVKATLSVIDAGATLITGEAGPLAFISCSECVTSRVGP
FG09874.1 	GIGIIRSLATGPPTDATQWIGTAVSLLVGIMDAGATLITGDEAPLAYLTCAEKVTERLGP
AN5840.1  	GCGLIQGLAAGPAIDADDWINPALASLLELSKAGAGLFAGDVISNAYIKCSEKVSSRLGP
          	* *:::.**:**. **  *:..*:   :   .*** *::*:  . *:: *:: *:.*:*.

MG04710.1 	FRQFIGVATLRAHGVELLPENLKAEPFED~LVTRVMYRLRFAGEQRPFDVVSVNYMLPLA
NCU05803.1	IRPFIGVATLRAHNVSALPENLTEEPFDD~LITRALYRLRFAGEQRPFDVISLIYMLPLI
FG09874.1 	MRPFVGIAALRLRGVS-LSENYQEEAVED~LITRVLYRLRFAGEQRPFDSVSLIYALPLV
AN5840.1  	LRQFVGVATLRAMGKGTVPAEMELEPLGK1LVTRILYRLRFASEQRPLDNSSLAYALPLL
          	:* *:*:*:**  .   :. :   *.. . *:** :******.****:*  *: * *** 

MG04710.1 	LLILNKGGFG-ATAEDRDTHLVLATELLSFHTDTA~SSEALPRAQMLETLISSMQVYNQH
NCU05803.1	LLVLEKGGFG-SNADDKDATLVLAIEFLSFHTDVY~ADEATPRAEILSTLITSMQNYNQH
FG09874.1 	LDLLRKGGVG-SSADDADAQLVLAIEFLSYHTDVC~ADEAVPRAELLSVLISSMQAYAQH
AN5840.1  	FTVLAQNGIEEEKGDEEGEQVLLALEILSYHSGSF1TDNRLPRVEILGQLISSMQRYTQH
          	: :* :.*. . ..:: .  ::** *:**:*:.   :.:  **.::*  **:*** * **

MG04710.1 	YKIIRDCFSDMVRCVAPNISEKEIGVVARGAIVPQTSVRTTVLQSISADVDMSELEFSEE
NCU05803.1	YKIIKDCFSDMVRCIAPNISAEEIAVLSRGAIVPQVAVRTAALQAISSDVDMSELSTSEE
FG09874.1 	YKLLKDCFADMCRCIAPNMDQEEMVVLAKGTLVPETRVRSTVLQSISAEVDMSDLGYSDE
AN5840.1  	YKLIKDTLFDASRCIAPNINENEVNVLLKGTIVSDPSVRSAVLQAIDAEIDLTDMDFSEH
          	**:::* : *  **:***:. :*: *: :*::*.:  **::.**:*.:::*::::  *:.

MG04710.1 	IWIAYHEDSEENVELAKEIWEESGFQTSKDVPVKMLPYLESKDGQLRKAASRALAEACSN
NCU05803.1	IWLACHDDVPENADLGRDIWEESEFQVTEELAFKMLPYLESKDGQLRRAAAKGLAEALGQ
FG09874.1 	IWVAAHDDEEENQDLGREIWEESGFEVTADMPLRMLPFLESKDGQLRRSAARSLAEAVSL
AN5840.1  	IWLECHDHVEENAEIAESIWEENGLEIDETAYGKLIPYLDSRDSNLRGAAARAIAHAIES
          	**:  *:.  ** ::...****. ::       :::*:*:*:*.:** :*::.:*.*   

MG04710.1 	HPETVNLILEKLRLAYVEFAKPRVPELDEFGMPKKMDLSDPWEARHGIASSFKELAPYLK
NCU05803.1	NLSAVNPILEKLRESYTELAKPRLPELDEFGMPKKKDLSDPWEARQGLALAFQGIAPLLQ
FG09874.1 	YHEALEDVLEQLKSTYIELAKPRVQQLDEFGMPKKMDLSDPWEGRQGIATAFRELASVIT
AN5840.1  	NHSVFGDIISQLQSKYEFEIRPKEPEKDKYGMPKKVDTTDHWESRSGIALAFGAMQNLFE
          	  ...  ::.:*:  *    :*:  : *::***** * :* **.* *:* :*  :   : 

MG04710.1 	REHLDSFFAFLIEQGPLGDQNGSVRAEMLEAANKAIEIHGKGMVDKLMKTFETTLEAPDK
NCU05803.1	KNQLEPFFAFLIDNGPLGDQNGNVRAQMLEAANTAIEIHGKSILDKLMKTFEKTLEAPDK
FG09874.1 	ADQLDPLFDFLINAGPLGDKNGAVRSEMLDASIKAIEIHGKTILDKLMSKFEQTLEQPDT
AN5840.1  	GDQIVSFLRFLIERGPLIDRSSVVRDQMADSGKGVIAARGQEKVEELMKLLETTLETSDK
          	 ::: .:: ***: *** *:.. ** :* ::.  .*  :*:  :::**. :* *** .*.

MG04710.1 	GSAAADRVNEAVIIMYGALARHLKAGDAKIPVVIERLIATLSTPSEAVQYAIAECLPPLV
NCU05803.1	GTAFADRVNEAVIIMYGALARHLKHGDAKIPVVIDRLIATLSTPSETVQYAIAECLPPLV
FG09874.1 	NSDAADRVNEAVIIMYGALARHLSPGDPKIPIVIDRLVATLSTPSETVQYAIAECLPPLI
AN5840.1  	GSETSDLLNEAVVVLYGSLAQHLKSDDPRLQTVIKRLLATLPTPSESVQSAVSDCLPPLI
          	.:  :* :****:::**:**:**. .*.::  **.**:***.****:** *:::*****:

MG04710.1 	KACGNKSSKYFDEIMETLLTSKNYAVQRGAAYGLAGLVLGRGIASLREFRIMSNLHSALE
NCU05803.1	RTCGDKSSKYIEQVIETLMTSKDYAVQRGAAYGLAGLILGRGVKSLKEHRILITLRSALE
FG09874.1 	QACPDQSSKYFGQILEQLLSSKKYAVQRGSAYGLAGLVMGRGIASLREYRILPTLNDAVE
AN5840.1  	RLAGRQSGKYAQEMLDQILQSKDYATQRGAAYGLAGIVKGRGIVTLREFRVMPHLQDAAE
          	: .  :*.**  :::: :: **.**.***:******:: ***: :*:*.*::  *..* *

MG04710.1 	NKKEANQRESALLAYELLATILGRLFEPYVILIVPQLLAGFGDSNANVRDAALASAKACF
NCU05803.1	NKKEVNQRESAMIALELLSTILGRLFEPYVIQIVPQLLAGFGDSNPNVREAALHAAKSCF
FG09874.1 	NKKEANQREAALLAYELLATMLGRIFEPYVIQIVPQLLTGFGDANANVRDACLAAAKACF
AN5840.1  	NKKEPHKRLGALMAYELFATILGRTYEPYVIQVVPQLLTSFGDPNADVREACLYTAKACF
          	**** ::* .*::* **::*:*** :***** :*****:.***.*.:**:*.* :**:**

MG04710.1 	ARLSSYGVKQILPTLLRGLDDDQWRSKKGACDLLGAMAYLDPQQLALSLPEIIPPLTAVL
NCU05803.1	AKLSSFGVKKILPTLLDGLDEDQWRSKKGACDLLGAMAYLDPQQLAQSLPEIIPPLTAVL
FG09874.1 	ARLSSYGVKKIMPTLLNGLEEQQWRSKKGACDLLGAMAYLDPQQLANSLPDIIPPLTGVL
AN5840.1  	SNLSSYGVKKILPTLLEGLDDTQWRSQKGACDLLGAMAYLDPQQLAVSLPEIIPPLTVVL
          	:.***:***:*:**** **:: ****:******************* ***:****** **

MG04710.1 	NDSHKEVRSGANKSLKRFGEVISNPEVKGLVDILLKALSDPTKYTDEALDSLIKVQFVHY
NCU05803.1	NDSHKEVRLAANKSLKRFGEVIENPEIHSLVDILLKALSDPTKYTDEALDALIKVQFVHY
FG09874.1 	NDSHKEVRAAANRSLKRFGEVINNPEIKSLVDIILKALSDPTKYTDEALDSLIKVQFVHY
AN5840.1  	NDTHKEVRNAANRSLQRFGEVISNPEIKSLVNVLLKALSDPTKFTDEALDALIKVSFVHY
          	**:***** .**:**:******.***::.**:::*********:******:****.****

MG04710.1 	LDAPSLALVSRILQRGLADRSNTKRKASQVIGSLAHLTERKDLVSHLPVLVAGLKIAVVD
NCU05803.1	LDAPSLALVSRILQRGLGDRSNTKRKAAQVIGSLAHLTERKDLVAHLPVLVAGLKIAIVD
FG09874.1 	LDAPSLALVTRILQRGLADRSNTKRKAAQVIGSLAHLTEKKDVIMHLPVLVAGLKLAVVD
AN5840.1  	LDAPSLALVVRILERGLGDRSATKRKSAQIIGSLAHLTERKDLITHLPIIVSGLNLAIVD
          	********* ***:***.*** ****::*:*********:**:: ***::*:**::*:**

MG04710.1 	PVPTTRATASRALGSLMEKLGEEALPDLIPGLMQTLKSDTGAGDRLGSAQALSEVLAGLG
NCU05803.1	PVPTTRATASRALGSLVEKLGEDALPDLIPGLMQTLKSDTGAGDRLGSAQALSEVLAGLG
FG09874.1 	PVPTTRATASRALGSLVEKLGEDTLPDLIPGLMQTLKSDTGAGDRLGSAQALSEVLAGLG
AN5840.1  	PVPTTRATASKALGSLIEKLGEDALPDLIPNLMSTLKSDTGAGDRLGSAQALSEVLAGLG
          	**********:*****:*****::******.**.**************************

MG04710.1 	TTRLEETLPTILQNVESSKPAVREGFMSLFIFLPVCFGNSFANYLGRIIPPILSGLADDI
NCU05803.1	TSRLEETLPTILQNVESSKPAVREGFMSLFIFLPVCFGNSFANYLAKIIPPILSGLADDA
FG09874.1 	TTRLEETLPTILQNVESSKPAVREGFMSLFIFLPVCFGNSFSNYLGRIVPPILAGLADDI
AN5840.1  	TTRLEETLPTILQNVSSSKATIREGFMTLFIYLPACFGNSFAPYLGRIIPPILAGLADDV
          	*:*************.***.::*****:***:**.******: **.:*:****:***** 

MG04710.1 	EAIRETALKAGRLLVKNFAVRAVDLLLPELERGLADDSYRIRLSSVELVGDLLFNLTGIS
NCU05803.1	ESIRETALRAGRLLVKNFAVRAVDLLLPELERGLADDSYRIRLSSVELVGDLLFNLTGVK
FG09874.1 	ESIRETALRAGRLLVKNFAARAVDLLLPELERGLADDSYRIRLSSVELVGDLLFNLTGIK
AN5840.1  	DTIRETSLRAGRLLVKNFSSKAIDLLLPELERGLADDSYRIRLSSVELVGDLLFSITGIT
          	::****:*:*********: :*:*******************************.:**:.

MG04710.1 	GKTEDGDEDEEEKVKEAGNSLREALGDEKRNKILSALYICRCDTATSVRAAAVAVWKALV
NCU05803.1	VTDGEDEEEDIETVKEAGASLREILGEEKRNKVLSSLYICRCDTSGAVRSAAVSVWKALV
FG09874.1 	AGTEAEDIEEDENIKEAGASLKETLGEDKRNKILSALYVCRCDTAGAVRSAAIAVWKVLV
AN5840.1  	AKTEAEEEDEE--AAQAGQSLLEVLGEERRDKVLSALFICRCDTSGLVKTAAMGVWKSLV
          	      : ::     :** ** * **:::*:*:**:*::*****:  *::**:.*** **

MG04710.1 	SSPKTLKELVPTLTQLIIRRLGSTNMEHKVIASNALGELIRKAGDNVLSSLLPTLEEGLQ
NCU05803.1	HSPRILKELVPTLTQLIIRRLGSSNMEHKIIASNALGELIRKAGDGVLATLLPTLEEGLQ
FG09874.1 	HSPRTLKELVPTLTQLLIRRLGSSNMEHKVIASNALGELIRKAGDGVLSSLLPTLEEGLQ
AN5840.1  	ASPRTLKEMVPTLSQLIIRRLGSSNMEQKVIASNALGDLIKKAGESVLATLLPTLEDGLQ
          	 **: ***:****:**:******:***:*:*******:**:***:.**::******:***

MG04710.1 	TSTDVDARQGICLALKELISSASEEALEEHEKILISVVRTALTDSDTEVREAAAEAFDSL
NCU05803.1	TSSDVDARQGICLALKELIASASPEALEDHEKTLISVVRTALTDRDEDVREAAAEAFDSL
FG09874.1 	TSVDVDAKQGICLALRELISSASPEALEDHEKTLISVVRTALTDSDEDVREAAAEAFDSL
AN5840.1  	TSPDVDVKQGICIALKEIIASSSPDALEDYEKILISIVRVALVDNDDDVREAAAEAFDAL
          	** ***.:****:**:*:*:*:* :***::** ***:**.**.* * :**********:*

MG04710.1 	QQILGKRAVDQVLPFLLNLLRSEDEAENALSALLTLLTETTRSNIILPNLIPTLIAPPIS
NCU05803.1	QQILGKRAVEEVMPFLLTLLANEEEAENALAALLTLLTETTRSNMILPNLIPSLIKPPIS
FG09874.1 	QQIFGKRAVDQVLPFLLNLLRSEDEADNALQALLTLLTETTRSNIILPNLIPTLTTPPIS
AN5840.1  	QQILGKKAVDQVLPHLLLLLRNDEDAEQALSALLTLLTEQTRANIILPNLIPTLLTSPIS
          	***:**:**::*:*.** ** .:::*::** ******** **:*:*******:*  .***

MG04710.1 	AFNAKALASLSRVAGAAMNRRLPNIVNSLMDNLVNCKDDSLREDLDASFHTVISSIDEYD
NCU05803.1	SFNAKALASLSKVAGAAMNRRLPSIINSLMDNIINCTDDTLREELDESFDTVILSIDEYD
FG09874.1 	SFDAKALASLSKVAGPAMNRRLPNIINSLMDNEINCKEDGLREELATSFDTVIQSIDEYD
AN5840.1  	TFNAKALASLAEVAGSAMARRLPTILNTLMDNIITCKDDEQRKELDDAFSTILVSVDEFD
          	:*:*******:.***.** ****.*:*:**** :.*.:*  *::*  :* *:: *:**:*

MG04710.1 	GLNTVMNVLLQLTKHEDHRKRAATGKQLARFFAATDVDYSRYNQDIIRSLLISFDDSDME
NCU05803.1	GLNMVMQTLLQLLKHDDHRRRASTAFHLSKFFASADVDYSRYNQDIIRSLLLSFDDRDMD
FG09874.1 	GLNTVMNVLLQLLKHEDHRRRAATAYHMANFFAAASVDYSRYSQDIIRSLLNSFDDRDDG
AN5840.1  	GLNVMMSVMLALIKHDDERRRTRAADHLRRFFSDAEIDFSRYHQDLIRVLLISFDDPDKD
          	*** :*..:* * **:*.*:*: :. :: .**: :.:*:*** **:** ** **** *  

MG04710.1 	VVKSAWSALSEFTKKLRKEEMEALVVSTRQTLLQVGVAGNNLKGFELPKGVSAILPIFLQ
NCU05803.1	VVKSAWSALSEFTKKLRKEDMENLVISTRQTLLQVGVAGVNLRGFELPKGINAILPIFLQ
FG09874.1 	VVKAAWAALSAFTKKLRKEEMESLVISTRQTLQRVGVAGANLRGFELPKGINAVLPIFLQ
AN5840.1  	VVKAAWGALSGLTSRLRKEEMEVLSVPTRQILKGVGVPGADLPGFSLPKGITAILPIFLQ
          	***:**.*** :*.:****:** * :.*** *  ***.* :* **.****:.*:******

MG04710.1 	GLMNGTPDQKIQSALAISDIVARTSEASLKPFVVQITGPLIRVVSERSTEVKS~AILLTL
NCU05803.1	GLMNGSADQRVASALAISDIVDRTSEASLKPFVTQITGPLIRVVSERSTEVKS~AILLTL
FG09874.1 	GLMNGTADQRVQAALGISDIVDRTSEASLKPFVTQITGPLIRVVSERATEVKS~AILLTL
AN5840.1  	GLLNGSVEQRTQAALAIGDLIDRTAADSLKLFVTQITGPLIRVVSERSVDLKC1AIFFTL
          	**:**: :*:  :**.*.*:: **:  *** **.*************:.::*. **::**

MG04710.1 	NNLLEKMPTALKPFLPQLQRTFAKSLADTTSEQLRSRAAKALGTLIKYTPRVDPLIAE~L
NCU05803.1	NNLLEKMPTALKPFLPQLQRTFAKSLADTSSDVLRSRAAKALGTLIKFTPRVDPLIAE~L
FG09874.1 	NNLLDKMPTALKPFLPQLQRTFAKSLADPSSETLRTRAAKALGTLIKYTPRIDPLIAE~L
AN5840.1  	NKLLQKIPLAVKPFLPQLQRTFARGLADQTSETLRNRAAKGLGILITLTPRVDPLIAE1L
          	*:**:*:* *:************:.*** :*: **.****.** **. ***:****** *

MG04710.1 	VTGSKTTDPGVRTAMLKALFEVISKAGANMGEPSRAAVLGLIDMETDEKDD~AMTVTNAK
NCU05803.1	VTGSKTSDAGVKTAMLKALYEVISKAGANMGEGSRTAVLGLIDTEADERDD~TMTITYAK
FG09874.1 	VTGSKTADPGVKTAMLKALYEVISKAGANMGEASRASVLSLIDMDTDERDE~AMTITNAK
AN5840.1  	ITGTKTADVGVRNAMMKALQEVVGKAGANMSEASKNSLLALIDDDASDQTD1AVAITNAK
          	:**:**:* **:.**:*** **:.******.* *: ::*.*** ::.:: : ::::* **

MG04710.1 	LLGALVKNVSGDAAHNLIK~SRVLTPTPTTSSVLGLNAVLLDAPAAIMEGPFAEELPELL
NCU05803.1	LFGALVKNVSDEVAAQLLK~NRVLTRDFSNSSVLALNAVLLESPETLLESSLVDDLPELL
FG09874.1 	LLGALIKNVPEEAALGLLK~NRVVTPHFTHSSVLALNSVLAESPDALLQSSLVDDLPDLL
AN5840.1  	LLGALVKVLPPATAGPLIK2SRVLTSNFSHASILGLNALLVDSPSSLTEH-FAAETVNLI
          	*:***:* :.  .*  *:* .**:*   : :*:*.**::* ::* :: :  :. :  :*:

MG04710.1 	VQGISSKI0AMIADNSILAAGKYLLNEQSKPFEHTKAVFEALANTVGPGNPTDSRRLSLV
NCU05803.1	CQGMASKN0TFITENFILATGKYLLSPVPKSFEATKPLFSTISTLLPPGQPTDSRRLALV
FG09874.1 	CQGVINKN0VFVADNCILATGKYLLSDSAKTFETTKGIFEALASVIQPGNATDSRRLALV
AN5840.1  	CQGVTHKD0PFISDNSVLAAGKYLLLEEQHSFESNKAIFEALSPCLQPGTPSDTRRLALV
          	 **:  *   ::::* :**:*****    :.** .* :*.:::  : ** .:*:***:**

MG04710.1 	VVRTVSRVNMDLARPHLPQLAGPVFASVRDPVIPVKLAAEAAFVSLFNVADDESKVFDKF
NCU05803.1	LVRTLARTNPDLVRPHLALLAPPVFASVRDMVIPVKLAAEAAFVQLFAVADEESKVFEKY
FG09874.1 	VVRTVSRNDMEMVRPHVALLAQPIFASVRDPVIPVKLAAEAAFVELFNVADEESRIFDKF
AN5840.1  	VIRTVGRLHSELTRPHLGLLAPPIFAGVRDMVIPVKLAAEAAFLSIFSVVDSDSAVFDKY
          	::**:.* . ::.***:  ** *:**.*** ************:.:* *.*.:* :*:*:

MG04710.1 	MASPAGESMPANAKRSMQDYFKRVALRLGAQVRERREAEGGAGGLGLSNDEVEDEKEIWS
NCU05803.1	LESQEGN-MAPNVKRSMQDYFKRVALRLGAQVRERREAEGGTGGLGLSNDEVEDEKELMS
FG09874.1 	MAG-AGADLAPNTKRSMGDYFKRVAMRLGSQARERREAEGGQGGLGLSNDEAEDEKEIWS
AN5840.1  	MAG-PGAELPPGPKRSMSDYFKRVAMRLATQARERREAEGGQGGLGLSNDEVEDEKEVWS
          	: .  * .:... **** *******:**.:*.********* *********.*****: *

MG04710.1 	VGRIDSGED~VFG~KD------------------------------~-------------
NCU05803.1	VGRVDVAGG~GEM~FD------------------------------~-------------
FG09874.1 	VGKVDVGSE~AFT2CNAEKFWPCKPLSVFIQHPSRTRPSPLTTIKM2VNLRTQKRLASAV
AN5840.1  	IGKVELEEA1NHP~PRSQTPHFGNSIRCRVQRTSSANDRRYADIKM2VNLRTQKRLAASV
          	:*:::           :..    ..     . .: :     :  .   .  :..  ::: 

MG04710.1 	------------------------------------------------------------
NCU05803.1	------------------------------------------------------------
FG09874.1 	IGCGERKIWLDPNEQSEISNANSRQTIRKLISDGLIIRKPVTQHSRSRARELNLARREGR
AN5840.1  	VGCGKRKIWLDPNEMSEISNANSRQTIRKLVKDGLIIRKPVTMHSRSRARELNAARRIGR
          	 .... .   .... :. :.:.: .:  .  ...    .. :  : : : . . :   . 

MG04710.1 	------------------------------------------------------------
NCU05803.1	------------------------------------------------------------
FG09874.1 	HRGYGKRKGTADARMPSQVLWMRRLRVLRRLLVKYRASGKIDKHLYHELYHSSKGNAFKH
AN5840.1  	HRGLGKRKGTKEARMPSSLLWMRRMRVLRRLLVRYRAAGKIDKHLYHELYHLSKGNTFKH
          	  . .. ..: .:  .:.                  ::.. ..    .    :...: . 

MG04710.1 	-------~-------------------------------------------------
NCU05803.1	-------~-------------------------------------------------
FG09874.1 	KRALVEH0IHRAKAEKARETALQEEMDAKRAKNKAARERKQERAAAKRNALLAEE--
AN5840.1  	KRALIEH0IQKAKAERHRERILKEEMDAKRAKNKALRERRQERLEAKRNALVGEEQE
          	. :  .     :.:.   .   ... .:. :...:  .  ..   :. .:  .....
```
